# Carrying Out Rapid Qualitative Research During a Pandemic: Emerging Lessons From COVID-19

**DOI:** 10.1177/1049732320951526

**Published:** 2020-08-31

**Authors:** Cecilia Vindrola-Padros, Georgia Chisnall, Silvie Cooper, Anna Dowrick, Nehla Djellouli, Sophie Mulcahy Symmons, Sam Martin, Georgina Singleton, Samantha Vanderslott, Norha Vera, Ginger A. Johnson

**Affiliations:** 1 University College London, London, United Kingdom; 2 Royal College of Anaesthetists, London, United Kingdom; 3 Queen Mary University of London, London, United Kingdom; 4 University of Oxford, Oxford, United Kingdom; 5 King’s College London, London, United Kingdom; 6 The Australian National University, Canberra, Australia

**Keywords:** infectious, illness and disease, medical, anthropology, research design, methodology, qualitative, interviews, United Kingdom

## Abstract

Social scientists have a robust history of contributing to better understandings of and responses to disease outbreaks. The implementation of qualitative research in the context of infectious epidemics, however, continues to lag behind in the delivery, credibility, and timeliness of findings when compared with other research designs. The purpose of this article is to reflect on our experience of carrying out three research studies (a rapid appraisal, a qualitative study based on interviews, and a mixed-methods survey) aimed at exploring health care delivery in the context of COVID-19. We highlight the importance of qualitative data to inform evidence-based public health responses and provide a way forward to global research teams who wish to implement similar rapid qualitative studies. We reflect on the challenges of setting up research teams, obtaining ethical approval, collecting and analyzing data in real-time and sharing actionable findings.

## Background

Pandemics such as the COVID-19 outbreak, which began in December 2019, demand the timely sharing of not only epidemiological data but also research findings related to disease perception, social practices that might be linked to spread, health-seeking behaviors, health care delivery models, and barriers to care. Social scientists have a robust history of contributing to better understandings of and responses to infectious disease outbreaks and other emergency settings by providing this type of data (Henry, 2005; Hewlett et al., 2005; Koller et al., 2006; Koons, 2010). More recently, the work of social scientists during the Ebola outbreak in West Africa was actively, explicitly, and openly recruited by international outbreak response organizations such as the World Health Organization (WHO) and UNICEF (Abramowitz, 2014; Anoko, 2014; Fairhead, 2014; Ferme, 2014; Johnson & Vindrola-Padros, 2014; Richards & Mokuwa, 2014). The implementation of qualitative research in the context of infectious epidemics, however, continues to lag behind in the delivery, credibility, and timeliness of findings when compared with other research designs.

The authors form part of the Rapid Research Evaluation and Appraisal Lab (RREAL), a research team focused on the design and implementation of rapid qualitative research on health-related topics. The purpose of this article is to illustrate the rich history of rapid qualitative research during infectious disease epidemics, including our experiences of applying these principles to research on COVID-19. We are sharing the early findings of our work during the current pandemic to highlight the importance of qualitative data to inform evidence-based public health responses, and to provide a way forward for global research teams who wish to implement similar rapid qualitative studies.

### Using Qualitative Data to Inform Epidemic Response Efforts

An analysis of the engagement of social scientists in previous epidemics has pointed to a series of factors that influence when qualitative expertise is requested, how research is carried out, and how findings are shared to inform response efforts (Sams et al., 2017). In the case of the Ebola outbreak in West Africa, one of the key challenges faced by social scientists related to addressing the limitations of being asked to contribute to the response at a later stage in the outbreak (e.g., during or even after the epidemiological “peak” in some cases). Timeliness in forming research teams with the required expertise to collect data on the social determinants of disease is shaped by the stage when social scientists are offered a “seat at the table” (Martineau, 2015). Failure to include social science expertise in emergency planning operations limits the type of research that can be carried out (including the production of knowledge most relevant for response operations) and produces delays in the sharing of knowledge.

When offered a seat at the table, however, social scientists might still struggle to design and implement research in the context of an outbreak. For infectious epidemics and other types of complex health emergencies, qualitative research might not be allowed if deemed too intrusive or burdensome for research participants. Patients, health care workers (HCWs), public health authorities, or members of the public who are already struggling with the impact of the disease and delivery of health care response might not be able to assist with data collection or take part in studies. Furthermore, carrying out fieldwork during epidemics, where researchers often need to be in close contact with affected communities or health care facilities, exposes them to infection. Due to the immediacy of the situation, research in this context demands the sharing of findings in almost real time, requiring a type of data analysis that is not common in the social sciences. It also requires that “actionable” findings are shared. This refers to straightforward recommendations that can be easily understood and translated into changes in policy and/or practice and requires carefully planning the use of findings during the research design phase. Even if qualitative studies are produced during epidemics, public health officials might have difficulties trusting the findings, digesting the information and translating it into changes in policy and practice.

### Rapid Qualitative Research

Despite these limitations and potential challenges, rapid qualitative research approaches have been used to inform response efforts in the context of infectious epidemics and natural disasters since at least 2003. In a recent review, we found that rapid qualitative research is carried out to identify the causes of the outbreak, assess the infrastructure, control strategies, health needs, and health facility use (Johnson & Vindrola-Padros, 2017). Rapid qualitative research can be carried out in the difficult circumstances of an epidemic and provide findings that are timely and actionable (Abramowitz et al., 2015; Faye et al., 2015).

The field of rapid qualitative research is diverse and various design approaches have been developed, including rapid ethnographic assessments (REAs), rapid assessment procedures (RAPs), rapid assessment response and evaluation (RARE), rapid qualitative inquiry (RQI), rapid ethnographies (including quick, focused, and short-term ethnographies), and rapid evaluations, to name a few. McNall and Foster-Fishman (2007) produced an overarching definition of all Rapid Evaluation and Appraisal Methods (REAM) arguing that the features that all of these approaches had in common were (a) the study was conducted over a short timeframe (weeks or months), (b) the study design tended to be participatory, (c) the studies combined multiple research methods and triangulated data during data analysis, and (d) the studies were iterative, in the sense that data collection and analysis tended to be carried out in parallel and emerging findings shaped the data collection process (McNall & Foster-Fishman, 2007). Beebe (2014) has provided a similar definition of RQI as “intensive, team-based qualitative inquiry with (a) a focus on the insider’s or emic perspective, (b) using multiple sources and triangulation, and (c) using iterative data analysis and additional data collection to quickly develop a preliminary understanding of a situation” (p. 3).

There is a lack of consensus in relation to what is meant by “rapid,” with some authors arguing that rapid studies require 4 to 8 weeks (Scrimshaw & Hurtado, 1987), 90 days (Handwerker, 2001), or anywhere from a few days to 6 months (Vindrola-Padros & Vindrola-Padros, 2018). These time ranges are further complicated by rapid feedback and rapid cycle evaluations that might be longer in duration (perhaps 12 months) but include feedback or cycle loops as the evaluation is ongoing to share emerging findings. In addition to these rapid research approaches, some authors have also developed rapid techniques or tools for data collection and analysis that are used to reduce the amount of time required for specific research processes, such as speeding up interview transcription or the coding of qualitative data (Vindrola-Padros & Johnson, 2020). These techniques can be integrated into the rapid qualitative research approaches mentioned above or used in long-term research.

### Qualitative Research During the COVID-19 Pandemic

The current COVID-19 pandemic has produced a wide range of changes in our daily lives; changes which have been shaped by the attempts of the governments of countries around the world to limit physical interaction and reduce contagion. Research evidence has occupied a central stage in informing government policies, critiquing them, guiding clinical approaches for the diagnosis and treatment of COVID-19 positive patients, and exploring the social and economic impact of control measures (Fritz et al., 2020). Researchers have highlighted the importance of qualitative research, arguing that this approach can provide insight into aspects of behavior and perceptions often missed in epidemiological and clinical research as it allows us to “focus not just on ‘what’ but on ‘how’” (Teti et al., 2020). Qualitative research carried out during the COVID-19 pandemic can ask and answer questions which complement epidemiological data by providing insight into people’s lived experiences of disease, care, and epidemic response efforts (Teti et al., 2020). The exacerbation of social, health, and economic inequalities; the implementation of health care reorganization to address demands created by the pandemic; and the role and impact of different types of leadership at national and local levels can also be explored using qualitative research (Shah, 2020; Van Bavel et al., 2020).

Despite highlighting the benefits of carrying out qualitative research during the COVID-19 pandemic, few authors have discussed the challenges and practical issues faced when doing this type of research and doing it in a timely way. Our expertise in rapid qualitative research and infectious epidemics has meant that our team has been heavily involved in the implementation of rapid qualitative research to inform response efforts on COVID-19 at a local and global scale. In this article, we reflect on the barriers we have encountered and the strategies we have used to address them to share key lessons learned with other teams who might be considering producing and using qualitative data to inform pandemic response efforts (now or in the future).

## Method

This article draws from our experience with three ongoing research studies, each aimed at exploring health care delivery in the context of COVID-19. The three studies outlined below have different research designs: a rapid appraisal of HCWs’ perceptions and experiences, a rapid qualitative study using in-depth interviews on the use of qualitative data during infectious epidemics (including real-time data on COVID-19 as well as previous epidemics), and a mixed-methods survey of the impact of COVID-19 on the global delivery of cancer treatment during the pandemic.

### Study 1: A Rapid Appraisal of HCWs’ Perceptions and Experiences With COVID-19 in the United Kingdom and “Mirror Studies,” at a Global Scale

Previous qualitative research conducted with HCWs highlights the importance of understanding their personal experiences in providing care during periods of extreme crises, uncertainty, and where patient deaths are anticipated (Greenberg et al., 2020; Ives et al., 2009; Kobler et al., 2020). This rapid appraisal of frontline HCWs’ perceptions and experiences with COVID-19 comprises three streams: a policy review, media analysis, and telephone interviews with HCWs in the United Kingdom (see Table 1). Following a rapid appraisal design, this study was developed to collect and analyze data in an iterative way (Beebe, 1995).

**Table 1.** Study 1 Design: Data Collection, Sampling, and Data Analysis (Study in the United Kingdom).

Data Source	Method of Data Collection	Sample	Method of Data Analysis
Policy review	Policies were selected from legislation.gov.uk, gov.uk, National Health Service England (NHSE), and Public Health England (PHE) databases.	35 policies published between December 1, 2019, and April 20, 2020.	Data were extracted into Excel by one researcher and cross-checked by a second researcher who created a conceptual framework to categorize the policies.
Media analysis	Review of newspaper articles obtained from LexisNexis.	101 newspaper articles published between December 1, 2019, and April 20, 2020.	Data extracted using REDCap and analyzed for content using framework analysis (coding carried out by two researchers).
	Data were selected using the software “Meltwater” and sorted into pre-established categories.	146,000 social media posts were collected from the period between December 1, 2019, and April 30, 2020.	Social media content was analyzed using inclusion and exclusion framework, and coded the selected posts independently.
Frontline staff interviews	In-depth, semi-structured telephone interviews with a purposive sample of staff.	130 staff members working in emergency departments and intensive care units in three hospitals (doctors, nurses, and allied health professionals with different levels of training and expertise).	RAP sheets were used to synthesize findings on an ongoing basis. Selected transcripts were generated and analyzed using framework analysis.

*Note.* RAP = rapid assessment procedure.

#### Policy review

The policy review focused on health care policies to understand changes made to health care delivery in response to COVID-19 in the United Kingdom following the rapid evidence synthesis framework proposed by Tricco and colleagues (2017). U.K. Government policies were searched for, using the search strategy, databases, and inclusion criteria presented in Online Appendix 1.

#### Media analysis

A rapid media analysis was developed to capture perceptions and experiences of HCWs reported by them or third parties. Published newspaper articles were reviewed by running a series of searches on the Nexis database (see full strategy in Online Appendix 1 and findings in Table 2).

**Table 2.** Key Aspects of U.K. Newspaper Reporting of the Perceptions and Experiences of Health Care Workers With COVID-19.

Coverage in U.K. Newspapers	Overall		January		February		March	
*n* articles (row)	*N* = 50	100%	*n* = 1	2%	*n* = 7	14%	*n* = 43	86%
Key Issues Reported								
Insufficient advice/info/training	23	46	0	0	4	57.14	19	44.19
Adaption	23	46	0	0	1	14.29	22	51.16
Concerns over ability to cope	19	38	0	0	2	28.57	17	39.53
Personal protective equipment	18	36	1	100	0	0	17	39.53
Personal fears/family	17	34	0	0	1	14.29	17	39.53
Diagnostic resources	17	34	1	100	0	0	16	37.21
Contact tracing	8	16	0	0	3	42.86	5	11.63
Hospital infrastructure	14	28	0	0	1	14.29	13	30.23
Re-prioritization/Knock on effects	8	16	0	0	1	14.29	7	16.28

The social media analysis focused on Twitter but included relevant content from Reddit and publicly available groups and accounts on Facebook and Instagram which was posted from December 1, 2019. Meltwater, a media monitoring software, was used to conduct an English language Boolean query keyword search. The search terms used from the mass media analysis were adapted (details on the categories can be found in Online Appendix 1). Semantic discourse and topic analysis were used to understand the most frequent and weighted key words, hashtags and prioritized discussion themes, and clusters of topics within and across countries, primarily in the U.K. context (Van Dijk, 1985).

#### Interviews

Interviews were carried out with frontline staff from NHS hospitals in the United Kingdom. Interviews were semi-structured, focusing on HCWs’ views on the virus, patients, and the health care system organization and management. A purposive sample of 130 HCWs was selected for interview to cover a range of roles within the system (the full sampling framework can be found in Online Appendix 1). Interviews with staff are ongoing and will continue to contribute to emerging findings. While all interviews were audio-recorded, the main points were documented and compiled with real-time interview notes and further synthesized on a RAP sheet. RAP sheets are a tool commonly used in rapid qualitative research to summarize emerging findings so they can be shared while the study is ongoing (Beebe, 2014).

#### “Mirror studies.”

After the study was designed and approved in England, RREAL approached (or was approached by) other global research teams to determine whether they would be interested in replicating the study in their countries. The premise behind “mirror studies” was that each country would carry out the study independently, seeking local ethical approvals and managing data collection and analysis. RREAL helped facilitate the study setup by sharing our study protocol and study materials (information sheets, interview topic guide, consent form, etc.). All global teams have been in charge of making sure the findings from the studies can be used to inform local response efforts and published for academic audiences. The RREAL team will coordinate the synthesis of these published findings to create a global picture of the experiences of frontline staff during the COVID-19 pandemic. As the research is ongoing, we have also created a global virtual platform to bring all teams together to share information on the challenges of carrying out this research, and the strategies that have been used to overcome them. To date, the study is being replicated in 22 countries including United States, Mexico, Ecuador, Peru, Brazil, Chile, Argentina, France, Spain, United Kingdom, Ireland, Italy, Poland, Switzerland, Germany, Pakistan, India, Australia, South Africa, Nigeria, Democratic Republic of the Congo (DRC), and China.

*Ethical review*: The study was reviewed and approved by the Health Research Authority (HRA) in England (IRAS: 282069) as well as Research and Development (R&D) offices of the hospitals where the study took place. We followed an informed consent process.

### Study 2: A Rapid Qualitative Study on the Use of Qualitative Data During Infectious Epidemics

The aim of this study was to explore the use of qualitative data to inform epidemic response efforts and the barriers encountered when attempting to do so. This rapid study consulted a broad, diverse, and global participant base with experience of responding to epidemic outbreaks in any capacity, all of whom were involved in responding to the COVID-19 pandemic. Participants were sampled for telephone interviews using a range of purposive and snowball methods (i.e., recruiting through affiliated epidemic response networks, listservs, and those directed to the study by those who had participated).

Individuals consulted included fellow social scientists, community engagement workers, relief coordinators, frontline clinical staff, public health registrars, guideline creators, and program managers. They worked in the following geographical areas: West Africa (including Nigeria), DRC, Kenya, India, Bangladesh, United States, Italy, and the United Kingdom. Somewhat uniquely, this study was developed prior to the COVID-19 outbreak. It was originally intended to exclusively investigate low- and middle-income countries; however, following the outbreak of COVID-19 across the world, it was agreed that it was important to open up the sample to incorporate the experiences of those responding to the current pandemic, including those from high-income countries where outbreaks might be more acute and widespread (at the time of early data collection).

The study is based on telephone/online semi-structured interviews, all of which were audio-recorded and selectively transcribed. The interviews considered the main needs of individuals responding to epidemic outbreaks, how qualitative data were used in such circumstances (with consideration to data collection, communication, timeliness, and actionability), factors enabling/preventing the use of qualitative data (e.g., political, ethical, administrative, regulatory, or logistical factors), the potential impact of successful/unsuccessful qualitative data-use in epidemic outbreaks, and lessons learnt for future epidemics. The analysis utilizes a combination of narrative description and the framework method (Gale et al., 2013), for exploring the “qualitative data-use background” and developing themes in the “determinants” and “impacts” of qualitative data-use respectively.

*Ethical review*: The study was reviewed and approved by the UCL Ethics Committee (UCL REC): 6862/002. We followed an informed consent process.

### Study 3: A Mixed-Methods Survey of the Impact of COVID-19 on the Delivery of Cancer Treatment

The COVID-19 pandemic has affected the capacity of health care systems to deliver medical services for non-COVID-19-related conditions. Many areas of the world are reporting delays in cancer diagnosis or treatments having to be put on hold or reduced to emergency cases (Kutikov et al., 2020; Turaga & Girotra, 2020). There have been various strategies implemented at a national level and in local hospitals in an attempt to mitigate the risk of COVID-19 for cancer patients in active treatment. We developed a survey to explore the impact of COVID-19 on the delivery of cancer care, map the strategies being used around the world, and capture the learning generated in local hospitals. These findings will enable a better understanding of current measures, which will be important for informing care delivery in this pandemic and in future outbreaks.

The study was global, multidisciplinary, and cross-sectional. Qualitative and quantitative data were collected using a web-based survey instrument (Opinio 7.12). Both purposive and snowball-sampling techniques were employed to target oncology health care professionals. A multidisciplinary team of specialists and researchers developed a standardized survey. The survey questions were initially piloted within RREAL, and with clinician contacts of the principal investigator, to ensure content, language, length, and format were appropriate. The survey was refined following feedback from the pilot.

The survey was translated into Spanish and French and sent to a range of professional bodies including The International Society of Oncology Pharmacists, The U.K. Chemotherapy Board, The Clinical Oncology Society for Australia, and The Canadian Association of Pharmacy in Oncology. The professional bodies distributed the survey to their members by email link. The first page of the survey contained a description of the research, frequently asked questions, and a statement regarding consent to participate. Sample characteristics included country of practice, institution type, and professional role. The survey included a mixture of open-ended and closed-ended questions. The questions addressed the following: the current status of delivery of cancer care, the participant’s awareness of guidelines and policies concerning the prioritization and protection of patients receiving cancer care, the current strategies in place to prioritize and protect patients receiving this type of care, and the participants’ professional opinion of the strategies employed. The open-ended questions allowed us to collect qualitative data and these were particularly useful for identifying strategies used by hospitals to shield or protect cancer patients from COVID-19 additional to those offered in the survey. Participants were also able to reflect on the strategies they had considered to be the most effective. The last open-ended question in the survey asked participants if they had anything to add and several respondents used this to provide further reflection on their experience of delivering cancer care in the context of a pandemic. Participants’ responses were anonymous and data were securely stored on Opinio software. The survey results were summarized using descriptive statistics and the qualitative data obtained from free-text responses were analyzed using framework analysis performed in Excel (Gale et al., 2013). The analysis process entailed an unstructured familiarization phase, a coding phase initially framed by the survey questions but open to identifying new topics emerging in the data and a final coding phase to organize the data from all participants in the form of a table.

*Ethical review*: The study was reviewed and approved by the UCL Research Ethics Committee (UCL REC): 6862/005. We followed an informed consent process.

## Findings

We developed reflective cycles throughout the design and implementation of these studies, identifying our main concerns, problems we were facing, things we were doing well, and those we needed to improve. We documented these reflections in the form of notes, and we met as a team to discuss these data and decide on the main issues that needed to be included in this article. In this section, we discuss the main challenges that have emerged to date in the delivery of our three studies during the COVID-19 pandemic, and the strategies we have used to address them. We draw on conversations and decisions made within our research team as well as conversations with other global research teams, collaborators, ethical review boards, funding bodies, and R&D offices in local hospitals.

### To Research or Not to Research?

As with any type of study, the first question we asked ourselves when designing each study was, should we be carrying out research at this time? Would our research be burdening HCWs, public health authorities, or other members of staff who are already under immense pressure? Could our studies produce more harm than benefit? Our previous experience carrying out research in the context of infectious epidemics pointed to the importance of collecting data in real time and how prospective data collection would differ from retrospective, if we decided to carry out the study at a later date and based on participant recall. We knew that data collection and analysis would be difficult as we would have to consider not only the issues in relation to accessing participants but also the emotional impact this study could have on the researchers in the team. We also knew that if we wanted to make sure our research findings could be used to inform changes in policy and practice, we would need to establish collaborations with stakeholders to understand their evidence needs and timelines early on the process of designing the studies. We reached the conclusion that it would be unethical *not* to carry out the studies during the pandemic, as we would be missing relevant, immediate, and actionable information that could be used to inform local and global response efforts as well as preparedness strategies for future pandemics.

Despite moving forward with the studies, we were conscious of the fact that we would need to pay close attention to our study design to reduce potential research burden, limiting the amount of time we would require from staff. To account for this, we kept our interview guides brief (i.e., 15- to 20-minute telephone interviews), we carried out interviews at times of day most convenient for participants (including lunch breaks, nights, and weekends), and considered reducing the intensity of data collection at specific time points of the pandemic (i.e., during “epidemiologic peaks”). Our experience of recruiting staff to these studies has shown that, despite feeling overstretched, many HCWs wanted to take part in the study and have indicated that the interviews were a therapeutic process, where they could freely narrate their experiences to an external party and feel that their voice was heard.

Given that RREAL research designs are applied in structure—where findings are designed to be used by national and international organizations to inform response efforts—health staff have also indicated that taking part in the studies made them feel they were making a contribution beyond care delivery. Several participants spoke about being able to share lessons with other sites/countries and contribute to future learning for responding to disease outbreaks. Even though staff members were not expressing distress during the interviews or any indication that they were burdensome (in the case of the United Kingdom), several have indicated the importance of maintaining anonymity. All of our studies follow standard ethical processes for qualitative research, which guarantee the anonymity of participants and confidentiality of the data, and we have made this clear to participants in study materials and conversations before and after interviews.

### Ethical Review Processes

One important aspect of research setup we considered when thinking about the three studies was the ethical review and approval processes. Study 2 had been planned as a study before the COVID-19 pandemic began, so approvals for this study by our university research ethics committee (REC) were already in place. We did not have to make major changes to the study design as a result of COVID-19 but decided to expand the sample to include participants who had been involved in epidemic response efforts in high-income countries. We felt this would allow us to capture the experiences of some of the countries that were most affected during the most recent pandemic at the time of data collection. Study 3 also required review by a university research ethics committee, but, as it needed to be reviewed during the COVID-19 pandemic, changes to guidelines in relation to the prioritization of studies for review produced significant delays in the rollout of the survey.

An interesting experience worth mentioning in relation to Study 1 was a series of conversations that emerged when describing our study to other health services researchers and clinical colleagues and their automatic assumption that because the study was rapid, we would not be going through required ethical approval processes. This automatic association might be linked to the labeling of rapid research as a “quick and dirty” exercise (Vindrola-Padros, 2020b; Vindrola-Padros & Vindrola-Padros, 2018), or the belief that research that follows required processes will not be set up and implemented in time. We feel it is important to mention these conversations and situate them against the detailed ethical review processes described below to encourage research teams across the world to think differently about rapid qualitative research. As Beebe (1995) has argued, “rapid research” is not the same as “rushed research” and it can be carried out as rigorously as longer-term research studies.

Study 1 was based in England and required interviewing HCWs in the NHS. As a result, this study needed to be submitted to a centralized research authority board called the HRA following a relatively extensive bureaucratic process. After obtaining approval by this organization, the study would need to be reviewed and approved by the R&D offices of each hospital we hoped to recruit to the study. Fortunately for us, the HRA quickly set up a fast-track process for reviewing and approving studies on COVID-19. Our study was the first qualitative study to be approved as a fast-track study by this organization in England. A process that would normally take us several months was completed in a few weeks.

Securing R&D approvals was different and varied by hospital. While some R&Ds were able to assess their capacity to take part in the study quickly and issue an approval, others took longer and some even initially refused to process our request to take part in the study as it was not classified as a National Priority Urgent Public Health study. Only studies focused on vaccines, treatments, and diagnostic tests, and real-time collection of samples and data from people undergoing treatment could receive this classification (National Institute for Health Research, 2020)—limiting the research that can be carried out on the experiences and lessons learned by frontline HCWs (the focus of our study). This is an evident barrier to implementing rapid qualitative research on health services in the context of a pandemic in England. We regretted this decision and continue to find ways to make sure we can recruit the number of hospitals we originally sought to include in the study, but this new requirement might have a significant impact on our ability to document staff views and experiences and how these might differ by context.

The “mirror studies” described as part of Study 1 were dependent on each team managing the ethical review and approval processes required in their countries. Not all countries had established fast-track systems like the one described for England, and some countries relied on paper-based models that were put on hold during lockdowns. RECs were meeting remotely in some cases, but this was often less frequently. Although most teams identified this as a source of concern and potential barrier during early stages of the project, all teams were able to secure the required approvals.

Study 3 was submitted for ethical review by a university ethics committee during the COVID-19 pandemic. The university had set up a fast-track review process for all COVID-19-related research. Unfortunately, changes in guidelines for this fast-track review meant our application was put on hold for the first 2 weeks after submission as it had not been assessed by a senior member within the university. The application was further delayed by conversations with the ethics committee in relation to our sampling strategy, the scope of the study, and the dissemination of study findings. The study was approved 1 month after submission and we feel this was only as a result of our constant (sometimes daily) reminder that this was a time-sensitive study.

### Building of Research Teams and Funding

Rapid qualitative research demands the rapid setup of teams and sources of funding. Some countries have published calls for COVID-19 research, giving researchers much-needed resources to increase the capacity of their teams. Other teams, like ours, do not have external funding sources (which are not tied to other projects), and this led us to be creative in the design of our rapid COVID-19 studies, distribution of workloads, and types of partnerships and collaborations established with other research teams.

To adapt to these needs, we have utilized rapid review and systematized processes for documentary data, such as media analysis and policy reviews, as these approaches reduce the number of researchers and time required for collection, cross-checking, and analysis of evidence (Tricco et al., 2017). Following rapid analysis methods in the case of interviews, we have bypassed full interview transcription, and have analyzed data either directly from audio recordings or by using selected transcription (Neal et al., 2015; Vindrola-Padros & Johnson, 2020). Selected transcription was carried out internally by members of the team due to limited funds for sending out recordings for full transcription to a transcription company. The selected transcription was helpful for analyses where we knew we wanted to focus on specific topics, but members of the team highlighted that having full transcripts would have allowed them to get a better sense of the complete narrative of frontline staff.

Our team has the advantage of more than 13 years of experience in the field of rapid qualitative research and involvement in informing response efforts in previous infectious disease outbreaks. However, in the case of Study 1, this is the largest team we have coordinated to carry out rapid research, and it is mainly composed of students at the MSc and PhD level who either have volunteered their time to contribute to the research or are using the research findings as part of their theses or dissertations. One way in which we have addressed the issue of different levels of expertise has been by assigning “leads” to the specific analyses we are carrying out to bring together findings from the policy reviews, media analysis, and interview data. These specific analyses are currently focusing on the main areas of concern identified by frontline staff (e.g., well-being and mental health, personal protective equipment, end of life care, the impact on the wider health care system, and gender inequalities, among others). The leads assigned to each analysis are researchers with expertise on these topics either within our team or external partners who have quickly “upskilled” and provide ongoing support to more junior researchers.

### Data Collection and Analysis in Parallel to Share Emerging Findings in “Real-Time”

**Figure 1.**
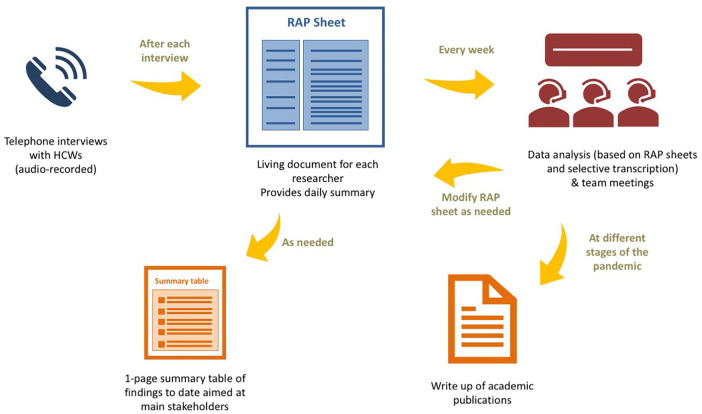
Process used for iterative data collection, analysis, and sharing of findings. *Note.* HCW = health care worker; RAP = rapid assessment procedure.

**Figure 2.**
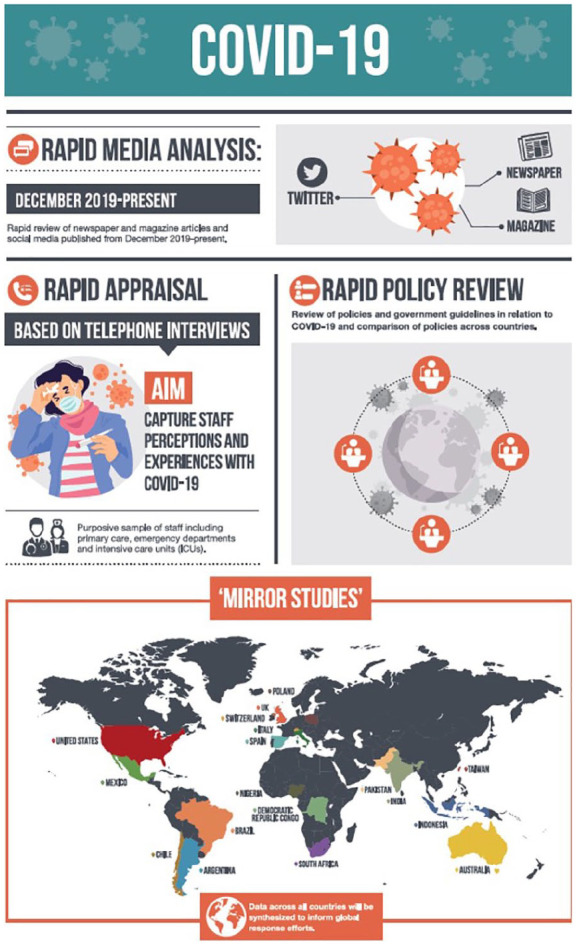
Example of infographic used to advertise Study 1.

A central component of the three studies has been the timely sharing of findings so they could be used to inform decision making and inform changes in practice. For Study 1, there was a period during the peak time of the pandemic in the United Kingdom that we were sharing findings bi-weekly with professional organizations in charge of redesigning care delivery in acute care hospitals. This rapid turnaround of findings was facilitated by intensive rapid techniques to facilitate the collection and analysis of data in parallel. In the case of the telephone interviews, these were audio-recorded by the interviewers who also took notes of the main topics discussed during the interviews. After each interview, the interviewers summarized these notes in the form of a table called a RAP sheet. The RAP sheet acted as a working document for each researcher. As new data were collected, the main findings were added to the RAP sheet. As a result, at the end of each day, each researcher had a summary of the main findings from the study obtained to date that could be further refined and shared with our primary stakeholders. The findings were not shared in an extensive report, but in the form of a one-page table (see Figure 1 for a description of this process). We also developed an infographic to disseminate the study design and will be using it to share emerging findings (Figure 2). We used a similar approach in Study 2 and shared this technique with countries taking part in the “mirror studies.”

As mentioned earlier, Study 3 used an online survey design to collect quantitative and qualitative data. Data collection and analysis were also carried out in parallel in this case as we started with analysis of the qualitative data as soon as we started receiving survey responses. This required frequent team meetings that happened weekly during more intensive stages. This early analysis allowed us to develop a coding framework that could be used by three researchers for the analysis of qualitative data.

The extent to which findings will be used to inform response efforts will depend on the research team’s capacity to engage with stakeholders. In our experience, it is always better if this is done early on in the research, not only to make sure the study is aimed at generating relevant findings but also to understand when findings are needed (Vindrola-Padros, 2020). In the case of all three studies, stakeholders were involved from the design of the research questions and remained a central component of the studies throughout all stages of the research. The type and frequency of findings they required changed through time and our team needed to be flexible enough to adapt to these changes.

## Discussion

In this article, we have sought to identify the most salient practical issues faced when carrying out three rapid qualitative studies during the COVID-19 pandemic. Our experiences have indicated that it is possible to implement rigorous qualitative research and deliver findings at a time when they can be used to inform changes in policy and practice. One of the first questions we faced in all three studies was deciding if we should carry out research during the pandemic. We agreed as a team that some data would always be better than no data and prospective research could capture snapshots of experience and meaning and how these changed throughout the pandemic. We did this acknowledging that there would be inherent limitations in relation to the data we could collect. We also knew that rapid qualitative research, if carried out well and responsibly, could do more good than harm if carried out before, during, and after a pandemic—but only if we were able to engage with stakeholders and share findings at a time and in a format to facilitate their use in decision-making processes. We were able to share findings at key time points because we used a series of techniques and tools commonly used in rapid qualitative research and rapid evidence synthesis.

To cover lots of ground in a speedy way, we relied on the work of a large group of researchers with different interests and levels of expertise. Each researcher made important contributions to the study, but the establishment of collaborations with other teams and incorporation of new researchers almost on an ongoing basis throughout the study demanded that we spend considerable time and energy on administrative and coordination tasks. It also meant that some researchers might have felt that they had to take on new responsibilities without feeling fully trained or prepared.

One of the main barriers in the implementation of rapid qualitative research experienced by our teams and other teams participating in the “mirror studies” were processes established by ethical review committees. In a recent publication, we have discussed proposals made by other researchers to establish separate ethical review processes for research that is deemed to be time-sensitive (Vindrola-Padros, 2020). For instance, a framework has been proposed by Tansey and colleagues (2010), for research on emergencies, where ethical approvals need to be obtained quickly. The authors have argued that this framework requires a combination of speed, depth, and proportionality (Tansey et al., 2010). The Ethics Review Board (ERB), an independent ethics committee that reviews studies carried out by non-governmental organizations such as Médecins Sans Frontieres (MSF) that can be considered time-sensitive, has also established its own ethical review framework (Schopper et al., 2009).

Numerous authors have argued that ethical review processes in universities and hospitals are not designed to adequately assess qualitative studies (Stevenson et al., 2015). Our experience carrying out rapid qualitative research during a pandemic has highlighted that some committees were able to develop fast-track processes that allowed us to begin research in a timely way, but ethical review still represented an important bureaucratic burden for our team and needed to be followed-up quite aggressively by our team leads. One way forward, even after the pandemic has ended, could be for ethical review committees in universities to analyze and learn from the processes used by committees used to working with time-sensitive topics such as the ERB mentioned above, instead of having to reactively improvise their own processes (some requiring complex prioritization processes such as the ones we faced in Study 3). Now that fast-track processes have been established for COVID-19 studies, another pressing question is the extent to which some of these could remain for rapid qualitative research that needs to be carried out after the pandemic ends (and for future health emergencies, in general).

In addition to the practical issues discussed so far, the experience of carrying out research during a pandemic allowed us to reflect on the value of the research we do and our responsibilities as researchers. The discussions we had with other research teams when attempting to establish collaborations for our study in the United Kingdom as well as the mirror studies in other countries pointed to the dominant perception of rapid qualitative research as low-quality or rushed research, as mentioned above. Quick associations were made between the length of the study, the extent to which the study would be reviewed by an ethical committee, the burden that would be placed on study participants, and the validity of the data collected using rapid study designs. Several authors have demonstrated that rapid qualitative research can be designed and carried out in a rigorous way (Beebe, 1995, 2014; Sangaramoorthy & Kroeger, 2020; Vindrola-Padros & Vindrola-Padros, 2018). We have also argued in favor of the need to define and describe methodologies rigorously, as well as outline how findings are used (Johnson & Vindrola-Padros, 2017). We have also proposed the development of reporting and assessment standards that can take into account the unique characteristics and challenges of these types of approaches (Vindrola-Padros, 2020b). These standards could be helpful for teams attempting to carry out rapid research under the pressure of a pandemic like COVID-19 or for those who find themselves experimenting with rapid techniques with no prior experience in this field.

The associations between the length of the study and the quality of the data might be the product of lack of familiarity with this body of literature. However, an issue to highlight is the fact that timeliness is not included in our definition of research and this has implications in relation to our responsibility toward the topics we study and participants who share their stories with us. If we are able to generate high-quality, timely findings during a global pandemic so they can be used to inform emergency response efforts, then should it not be our responsibility to do so?

In addition to the benefits already discussed in this article, rapid qualitative research also has limitations. Our studies have been able to capture a snapshot of a pandemic that will cause tangible long-term effects on the health of populations and their health care systems. However, questions remain in relation to the medical needs of patients recovering from the disease, the effects of the pandemic on the mental health of HCWs, the effects of the pandemic on other (non-COVID-19-related) medical services, its financial impact, and the extent to which some aspects of physical distancing will become “the new normal” in social interaction and work routines.

Our study designs might also be interpreted as instrumental in the sense that all studies sought to produce findings that could be used to make changes to policy and practice, in the first instance, and considered the production of knowledge of interest to academic audiences as a secondary aim. This might have limited our engagement with theory during initial stages of study design and implementation (although we have drawn from learning and conceptual frameworks from previous epidemics). In other words, we sought to reach a balance between (a) the production of analyses that might advance our conceptualization of theory and practice in light of the extreme pressures of a pandemic, (b) with more pragmatic analyses on the concerns and experiences of frontline staff and how these might be addressed in real time. In the face of what seemed to be a never-ending increase in deaths, the loss of loved ones and colleagues, and the witnessing of the raw realities of all of the HCWs who kindly shared their stories with us, we felt it was important to ensure our findings were timely and actionable. We hope our experiences can help inform the research conducted by teams who might be grappling with similar challenges around the world.

## Supplemental Material

sj-pdf-1-qhr-10.1177_1049732320951526 – Supplemental material for Carrying Out Rapid Qualitative Research During a Pandemic: Emerging Lessons From COVID-19 Click here for additional data file. Supplemental material, sj-pdf-1-qhr-10.1177_1049732320951526 for Carrying Out Rapid Qualitative Research During a Pandemic: Emerging Lessons From COVID-19 by Cecilia Vindrola-Padros, Georgia Chisnall, Silvie Cooper, Anna Dowrick, Nehla Djellouli, Sophie Mulcahy Symmons, Sam Martin, Georgina Singleton, Samantha Vanderslott, Norha Vera and Ginger A. Johnson in Qualitative Health Research
