# Supplementary material for: Carrying Out Rapid Qualitative Research During a Pandemic: Emerging Lessons From COVID-19
Source: Qual Health Res. 2020 Aug 31;30(14):2192–204. doi: 10.1177/1049732320951526 (PMC7649912; doi:10.1177/1049732320951526)
Supplement: sj-pdf-1-qhr-10.1177_1049732320951526 – Supplemental material for Carrying Out Rapid Qualitative Research During a Pandemic: Emerging Lessons From COVID-19 [file sj-pdf-1-qhr-10.1177_1049732320951526.pdf]

APPENDIX 1 – Study 1 additional information

RAPID MEDIA ANALYSIS

Search strategy for rapid analysis of newspaper articles (LexisNexis**):** ("healthcare professionals"[All Fields] OR "healthcare worker"[All Fields] OR "doctor"[All Fields] OR "nurse"[All Fields]) AND (("coronavirus"[MeSH Terms] OR "coronavirus"[All Fields]) OR corona[All Fields] OR ("COVID-19"[All Fields] OR "severe acute respiratory syndrome coronavirus 2"[Supplementary Concept] OR "severe acute respiratory syndrome coronavirus 2"[All Fields] OR "2019-nCoV"[All Fields] OR "SARS-CoV-2"[All Fields] OR "2019nCoV"[All Fields] AND ("coronavirus"[MeSH Terms] OR "coronavirus"[All Fields])) AND 2019/12[PDAT] : 2030[PDAT])))

The inclusion criteria for newspaper articles included in our analysis were:

1) Focus on the perspectives or experiences of healthcare workers (self-reported or narrated in third person);
2) Focus on the response strategies aimed at COVID-19;
3) Published from 1 December 2019 to 17 March 2020 (for the purpose of this paper); and
4) Published in English.

Data extraction and synthesis

All articles were screened by title and full text, and exclusions cross-checked. Disagreements were discussed until consensus was reached. The inclusion criteria used for article selection were: focus on the perspectives or experiences of healthcare workers (self-reported or in third person); focus on the response strategy for COVID-19; published from 1 December 2019 and published in English. Articles that met the inclusion criteria were analysed using a data extraction form developed in REDCap (Research Electronic Data Capture). The form was developed after initial screening of articles, piloted independently with a random sample of five articles before the form was finalised. Data were exported from REDCap and the main article topics synthesized. The information entered in free text boxes was exported from REDCap and analysed using framework analysis (Gale et al., 2013).

Search strategy for social media analysis (Meltwater):

Search terms

((bio:"healthcare professional" OR bio:"healthcare worker" OR bio:"doctor" OR bio:"NHS" OR bio:"nurse" OR bio:"physio*" OR bio:"Paramedic" OR bio:"Ambulance work*" OR bio:"Ambulance driver*") AND ("coronavirus" OR "#coronavirus" OR “corona” OR "COVID-19" OR "COVID 19" OR "COVID19" OR "#COVID19" OR "COVID_19" OR "COVID" OR "severe acute respiratory syndrome coronavirus 2" OR "severe acute respiratory syndrome coronavirus 2" OR "2019-nCoV" OR "SARS-CoV-2" OR "2019nCoV" OR "physio*" OR "PPE") OR

("i am" OR "as a" OR "source: I" OR "I'm a") near/5 ("doctor" OR "nurse" OR "doctors" OR "nurses" OR "Paramedic" OR "Ambulance worker" OR "Ambulance driver") AND ("coronavirus" OR "#coronavirus" OR “corona” OR "COVID-19" OR "COVID 19" OR "COVID19" OR "#COVID19" OR "COVID_19" OR "severe acute respiratory syndrome coronavirus 2" OR "severe acute respiratory syndrome coronavirus 2" OR "2019-nCoV" OR "SARS-CoV-2" OR "2019nCoV" OR "physio*" OR "PPE") NOT ("I am not" OR "I'm not"))

NOT ("RT" OR "QT")

Inclusion/Exclusion Criteria

To assess for predefined inclusion and exclusion criteria, imported news articles and news blogs use the URL's in Excel to access the full article. For social media data, the Hit Sentence was used to assess relevance and if they meet inclusion and exclusion criteria. For YouTube Media the URL was used to generate a transcription of the video and was screened for inclusion and exclusion data.

Inclusion Criteria

1. Content refers specifically to experience of Healthcare Workers (HCW) of healthcare delivery during the COVID-19 pandemic

Note: We are privileging first-hand accounts of experience but also included second-hand accounts if they refered directly to HCW experience of healthcare delivery.

Information source:

- HCW: first-had account
- HCW: second-hand account
  - Influencer (e.g. social media figure)
  - Public figure (e.g. celebrity, politician, academic)
  - Media story reference (news reporting)
  - Social media post reference
  - Public health or health organisation messaging
  - Government messaging
- Other

Exclusion Criteria

1. Post is by a non-HCW without specific knowledge of HCW experience;
2. Content is generally about health response and not HCW;
3. Content is generally about COVID-19 and not HCW;
4. Reference to HCW not associated with the COVID-19 pandemic;
5. Full text is unavailable (News article only);
6. Article is concerned with solely the financial market and will not be considered relevant to public sentiment;
7. News article duplicates from identical location, source and article information the duplicates are excluded. The first article sourced remained for inclusion in analysis.

Data extraction and coding

All tweets that met the inclusion criteria were coded into two pre-defined categories to create the final dataset. Code and inter-code reliability were checked in parallel to assess that divergence did not pass a pre-determined accuracy score. After initial coding, the dataset was cleaned for duplicates and semi-duplicates (e.g., posts retweeted with the prefix 'RT' or by a user/bot using random characters to avoid recognition by Twitter detection algorithms for mass postings).

Flow diagram of social media assessment:

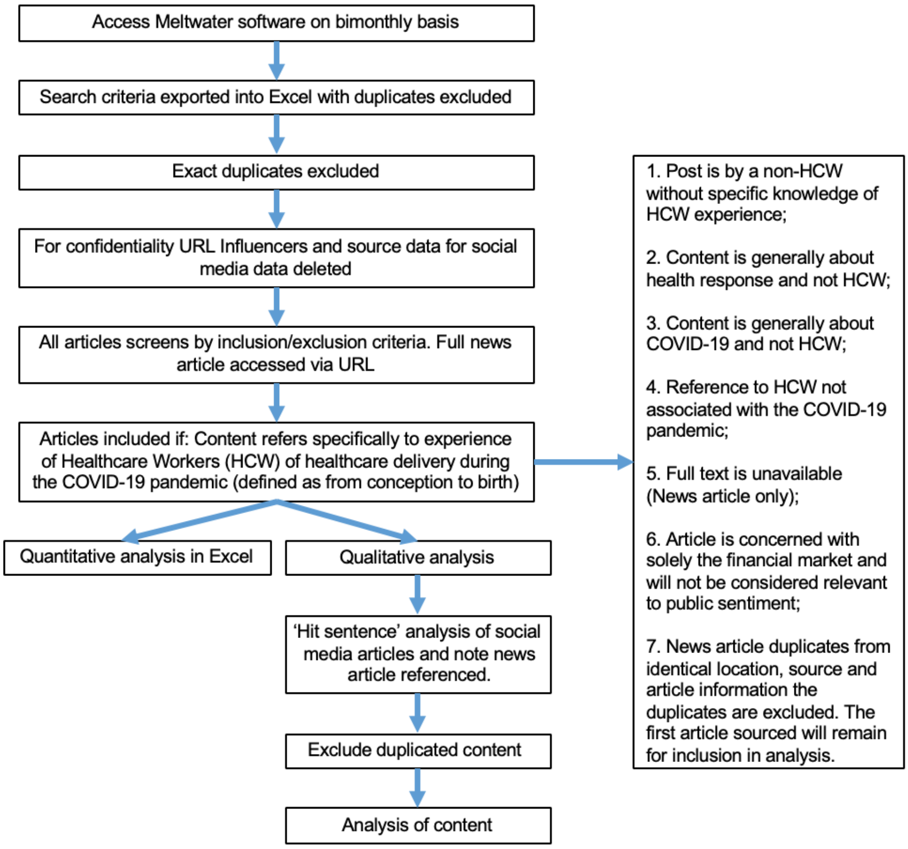

RAPID POLICY REVIEW

Search strategy for UK policy review: COVID-19 OR coronavirus OR corona.

Inclusion criteria:

1) Published from 1 December 2019 to 20 April 2020;
2) Aimed at healthcare delivery (i.e. not focusing on prevention, social isolation, etc.);
3) Related to the COVID-19 pandemic.

Data extraction and synthesis

All documents that met criteria were selected, and data were extracted into a spreadsheet. A reviewer with experience in health systems analysis identified the main topics that emerged from the data and developed a conceptual framework which was modified as new or amended policies were added to the analysis.
